# The Battle of LPS Clearance in Host Defense vs. Inflammatory Signaling

**DOI:** 10.3390/cells13181590

**Published:** 2024-09-21

**Authors:** Pankaj Kumar, Evan A. Schroder, Murugesan V. S. Rajaram, Edward N. Harris, Latha P. Ganesan

**Affiliations:** 1 Department of Internal Medicine, College of Medicine, The Ohio State University, Columbus, OH 43210, USA; kumar.953@osu.edu; 2 Department of Biochemistry, University of Nebraska, Lincoln, NE 68588, USA; eschrods1@gmail.com (E.A.S.); eharris5@unl.edu (E.N.H.); 3 Department of Microbial Infection and Immunity, College of Medicine, The Ohio State University, Columbus, OH 43210, USA; rajaram.3@osu.edu

**Keywords:** LPS, endotoxemia, scavenger receptors, clearance, signaling, liver, LSECs, Kupffer cells, sepsis, endotoxin associated diseases

## Abstract

Lipopolysaccharide (LPS) in blood circulation causes endotoxemia and is linked to various disease conditions. Current treatments focus on preventing LPS from interacting with its receptor Toll-like receptor 4 (TLR4) and reducing inflammation. However, our body has a natural defense mechanism: reticuloendothelial cells in the liver rapidly degrade and inactivate much of the circulating LPS within minutes. But this LPS clearance mechanism is not perfect. Excessive LPS that escape this clearance mechanism cause systemic inflammatory damage through TLR4. Despite its importance, the role of reticuloendothelial cells in LPS elimination is not well-studied, especially regarding the specific cells, receptors, and mechanisms involved. This gap hampers the development of effective therapies for endotoxemia and related diseases. This review consolidates the current understanding of LPS clearance, narrates known and explores potential mechanisms, and discusses the relationship between LPS clearance and LPS signaling. It also aims to highlight key insights that can guide the development of strategies to reduce circulating LPS by way of bolstering host defense mechanisms. Ultimately, we seek to provide a foundation for future research that could lead to innovative approaches for enhancing the body’s natural ability to clear LPS and thereby lower the risk of endotoxin-related inflammatory diseases, including sepsis.

## 1. Introduction

**Figure 1:**
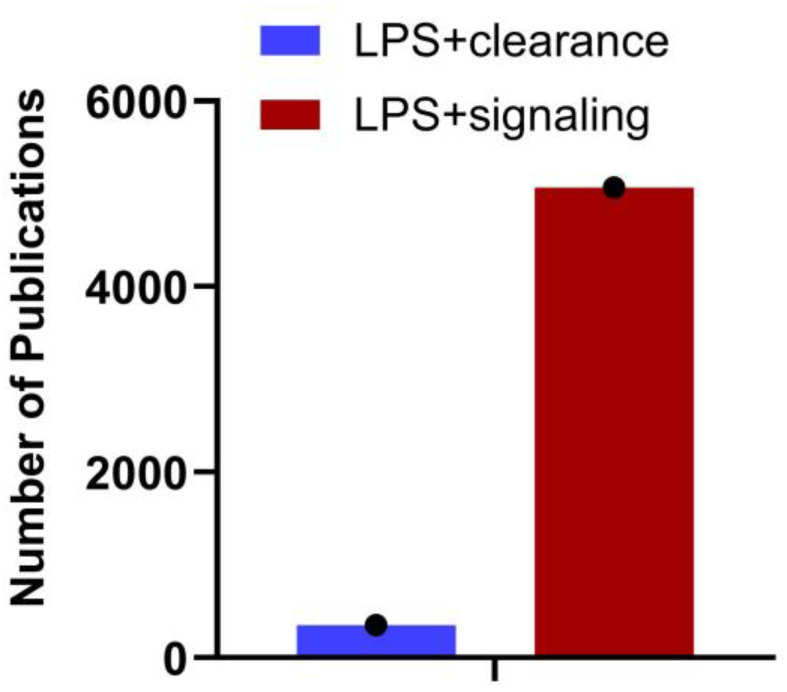
Number of articles published on LPS clearance vs. signaling. A search on NCBI using the keywords “LPS + clearance” and “LPS + signaling” reveals the number of articles published on each topic as of August 29th, 2024. The data underscores the importance of understanding LPS clearance.

Gram-negative bacteria discharge LPS/endotoxin into the bloodstream through their growth, cell lysis following antibiotic treatment, or translocation from the gut. The liver, which acquires blood from the gut via portal circulation, is the first major internal organ to encounter this LPS. The liver removes LPS from the bloodstream through endocytosis as part of the clearance process and metabolizes or detoxifies it. However, this process is not flawless, and some LPS can evade removal and continue circulating. LPS that evades hepatic clearance bind to TLR4 on various cells, including those in the liver and immune cells throughout the body. While the LPS-TLR4 pathway is well-characterized, the clearance of LPS and its impact on signaling require further investigation. The number of publications in PubMed (29th August 2024) with the search terms LPS + Clearance and LPS + Signaling (Figure 1) supports this. A detailed understanding of the immunological mechanisms governing hepatic clearance of LPS, and the subsequent systemic inflammatory responses, is essential for potential treatments and therapeutics. Since systemic clearance of LPS is directly related to its inflammatory effects, with reduced clearance leading to increased inflammation, bridging this gap will provide new insights into therapeutic strategies for modulating immune responses and treating diseases associated with endotoxemia. 

The immune system recognizes LPS as a pathogen-associated molecular pattern (PAMP), primarily through the pattern recognition receptor (PRR) TLR4, which is predominantly expressed on various myeloid cells and endothelial cells but is absent in lymphoid cells [1]. The binding of LPS to TLR4 leads to receptor dimerization and subsequent signaling activation [2]. Although TLR4 is expressed in all organs and circulating immune cells, quantitative studies in mouse models suggest that the spleen and lung are the primary sites where TLR4 expression is most prominent, surpassing that in the blood [3].

**Figure 2:**
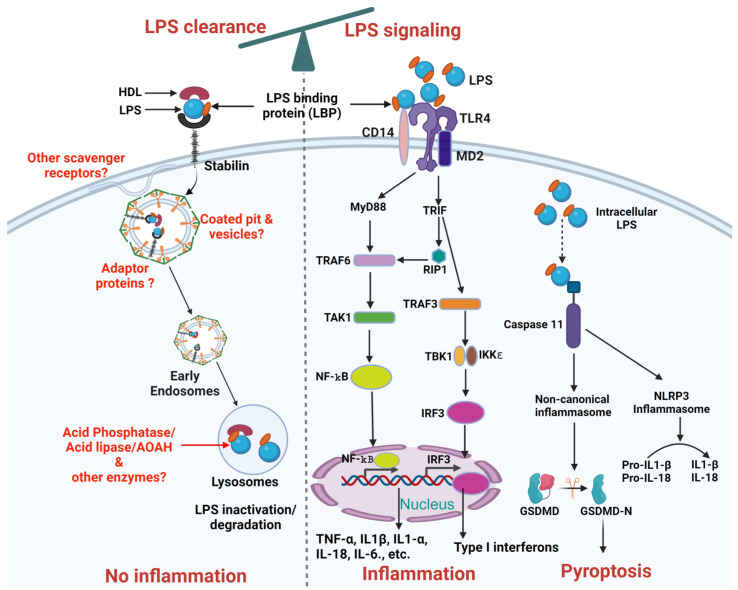
Schematic representation of LPS clearance and LPS mediated TLR4 signaling pathways. The balance at the top of the figure indicates the interdependence between LPS clearance and LPS signaling pathways. LPS Clearance: LPS binds to HDL facilitated by LBP, forming an LPS-HDL complex that enters the cell through Stabilin-1 and Stabilin-2 receptors mediated vesicular internalization, leading to inactivation/degradation by lysosomal enzymes. Future research needed is highlighted in red, including the identification of additional scavenger receptors, adaptor proteins, coated pits and vesicles, and other enzymes involved in LPS inactivation/degradation. LPS signaling: LPS stimulation in cells involves a series of interactions with several key proteins, including LBP, CD14, MD-2, TLR4. LBP acts as a carrier that binds directly to LPS and brings it to CD14. CD14 is a glycosylphosphatidylinositol-anchored protein that facilitates the transfer of LPS to the TLR4/MD-2 receptor complex. Subsequently, signals activated by TLR4 can be subdivided into MyD88-dependent, which occurs early and MyD88-independent, which occurs later and uses adaptors TRIF and TRAM. The MyD88-dependent pathway triggers through recruitment of TRAF6 which activates TAK1. TAK1 activates NF-κB by phosphorylation to inhibitory subunit IKKβ. NF-κB translocates to the nucleus and promotes transcription of pro-inflammatory genes (such as TNFα, IL-1β, IL1-α, IL-6, and IL-18). The MyD88-independent pathway depends on TRIF recruitment of RIP1 or TRAF3. TRAF3 activates IRF3 through TBK1 and induces transcription of type I interferons (IFNs) and IFN-inducible genes. Additionally, intracellular LPS either coming from endocytosed bacteria or leaked-out from the endosomes can activate caspase 11 dependent canonical or non-canonical inflammasome pathway. Intracellular LPS can bind to caspase 11 which can activate non-canonical inflammasomes and ultimately Pyroptosis. Similarly, caspase 11 can also activate NLRP3 inflammasomes through caspase 1 which can further proceed the conversion of pro-IL-1β and pro-IL-18 into active IL-1β and IL-18. Created on BioRender.com (accessed on 14 September 2024).

The liver, [4,5] including its non-parenchymal cells such as liver sinusoidal endothelial cells (LSECs) and Kupffer cells [6,7] and parenchymal cells, namely hepatocytes [8,9], was shown to be involved in LPS clearance. The LPS clearance pathway is mostly studied for how LPS is inactivated in the liver and the enzymes involved in detoxifying LPS. Macrophages modify lipid A by removing phosphate and acyl groups following phagocytosis [10,11] inactivating LPS. Few receptors have been demonstrated to clear LPS namely scavenger receptors [10] in general, and Stabilin [3] specifically, in addition to TLR4 [9] and low-density lipoprotein (LDL) receptors [12]. The roles of intra-cellular and extra-cellular proteins and lipoproteins in facilitating LPS clearance through interactions with these receptors remain unknown (Figure 2).

Furthermore, research on LPS clearance is relatively limited compared to LPS signaling, and the connection between LPS clearance and signaling has not been thoroughly explored, with only one study addressing this relationship. In that study, the authors demonstrated that higher LPS clearance via endocytosis is associated with reduced TLR4-mediated signaling [3]. These data suggest that by manipulating LPS clearance, LPS signaling and thereby, inflammation can be modulated, as illustrated schematically in Figure 2. This review aims to address this gap by summarizing the current literature on LPS clearance and highlighting the need for further research in this area. We also explore the interdependence between LPS clearance and LPS signaling, demonstrating how altering LPS clearance could potentially modulate signaling pathways and help control inflammatory responses. In the conclusion, we provide specific suggestions on how to upregulate LPS clearance to control TLR4-mediated inflammation, offering valuable strategies that could lead to novel therapeutic approaches for managing endotoxemia.

## 2. Endotoxemia

Endotoxemia refers to the presence of bacterial cell wall component LPS in blood circulation due to the shedding of bacterial LPS in plasma or due to increased gut permeability and high levels of intestinal LPS-containing bacteria [13]. Endotoxemia is associated with multiple disease conditions [14], the few that are listed here include sepsis [15], inflammatory bowel diseases [16], advanced liver disease [17], diabetes [18,19], chronic kidney disease [20], congenital heart diseases [21,22], atherosclerosis [23], Crohn’s disease [24], autism [25], and Alzheimer’s disease [26]. To treat endotoxemia, the ongoing strategy is to prevent LPS interactions with TLR4 and reduce inflammatory signaling [27].

LPS is highly toxic, with a maximum tolerated intravenous dose of only 1 to 4 ng/kg of body weight in humans [28]. The LD50, or median lethal dose varies among various species as reviewed by Warren et al. [29]. LPS can trigger strong immune responses in the host, leading to the production of pro-inflammatory cytokines such as tumor necrosis factor-alpha (TNF-α), interleukin-6 (IL-6), interleukin-1 (IL-1), and Interleukin-beta (IL-1β) [30,31,32].

**Figure 3:**
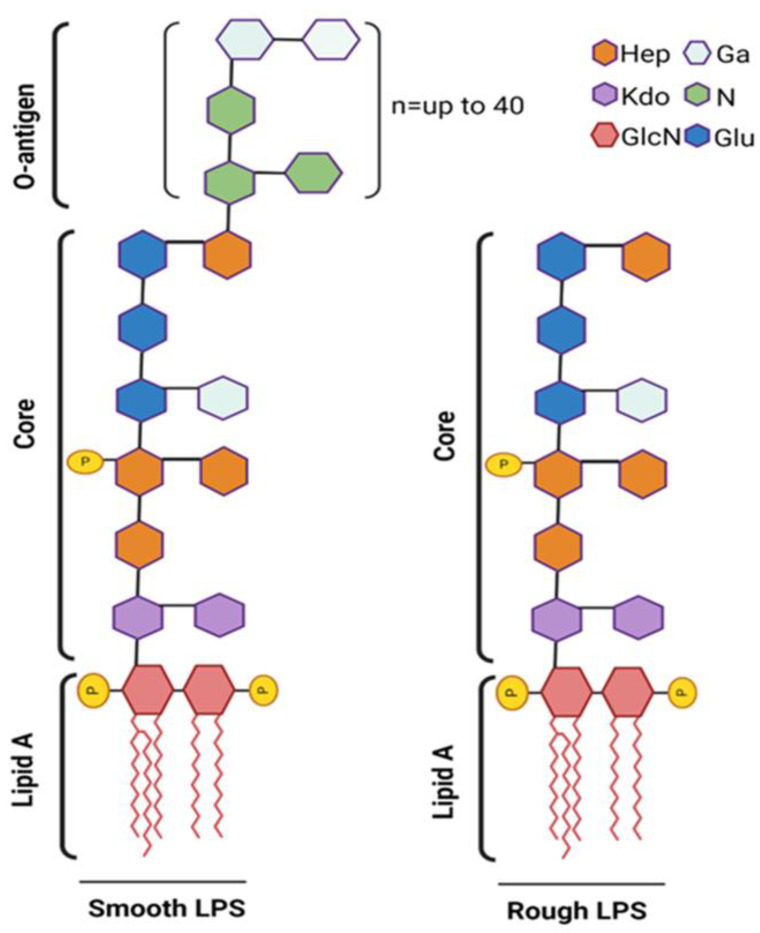
Schematic representation of smooth vs. rough LPS. The smooth LPS phenotype (**left**) expresses all three components: O-antigen, core oligosaccharide, and lipid A. The O-antigen is depicted as an extended chain attached to the core oligosaccharide. The rough LPS phenotype (**right**) lacks the O-antigen, showing only the core oligosaccharide and lipid A. Fatty acid chain length (n) and position may vary greatly among different species. Phosphate substitutions (P) are commonly found at C1 and C4′ of both GlcN (2-amino-2-deoxy-D-glucose) units that form the lipid-A moiety. Phosphate substitutions may also be found attached to core or O-antigen units. Created on BioRender.com (accessed on 14 September 2024).

Structurally, LPS is composed of the O-antigen, the core oligosaccharide, and the lipid A moiety, with the lipid A component being chiefly responsible for its endotoxic effects. LPS is classified into two main types based on the structure of its O-antigen region: smooth LPS and rough LPS (Figure 3).

## 3. Smooth LPS

Smooth LPS is characterized by the presence of a well-defined O-antigen polysaccharide chain, which extends from the core oligosaccharide region. The O-antigen is typically composed of repeating units of sugar molecules and imparts a smooth appearance to the bacterial cell surface under the microscope. Smooth LPS is often associated with virulent strains of bacteria and plays a key role in bacterial pathogenesis [33,34]. These immune responses are essential for the clearance of bacterial infections but can also contribute to the development of septic shock and systemic inflammatory response syndrome if dysregulated. The O-antigen region of smooth LPS is highly variable among different bacterial strains and is a major determinant of bacterial serotype specificity. This variability can impact the recognition of LPS by PRR such as TLR4 and influence the host immune response to bacterial infection [35].

## 4. Rough LPS

Rough LPS lacks a well-defined O-antigen region and is characterized by truncated or absent O-antigen polysaccharide chains [36]. As a result, the bacterial cell surface appears rough under the microscope. The absence of a complete O-antigen structure in rough LPS is derived from mutations in genes involved in O-antigen biosynthesis or modifications. While Gram-negative bacteria typically have smooth LPS in their natural environment, they become rough LPS mutants by losing their O side chains when isolated and cultured [37]. The selective advantage of preserving the smooth form of LPS, including the O side chains, in the natural environment is implied by the tendency of Gram-negative bacteria to lose these features when isolated [38]. Rough LPS has been shown to induce weaker immune cell responses and cytokine production in host cells. This reduced immunogenicity can be attributed to the lack of a complete O-antigen structure, which is essential for efficient recognition by host PRR. Even though it is less immunogenic, rough LPS may still trigger host immune responses and contribute to bacterial infections [39]. Overall, rough LPS can exhibit endotoxic effects and contribute to sepsis and septic shock, although to a lesser extent than smooth LPS, in vitro. Evidence suggests that their mechanisms of action may differ. For example, smooth LPS requires CD14 to activate both MyD88-dependent and -independent signaling pathways, especially at low doses. Rough LPS, on the other hand, can induce MyD88-dependent responses without CD14, even at low doses. However, in vivo, the differences in their ability to trigger innate immune responses are largely insignificant [39].

## 5. Sugars and Lipid Structures of LPS

The sugars and lipid structures of LPS play crucial roles in the functionality and immunogenicity of these molecules.

(i) Lipid A: Lipid A is the hydrophobic component of LPS and anchors the molecule in the outer membrane of Gram-negative bacteria [40]. Typically, it has a phosphorylated diglucosamine backbone with fatty acid chains attached to it. The number and length of the fatty acid chains can vary from 4 to 7 residues between bacterial species and can affect the biological activity of the LPS molecule [41]. Lipid A is responsible for the endotoxic activity of LPS, triggering immune responses in mammalian hosts [42].
(ii) Core Oligosaccharide: The core oligosaccharide is a short chain of sugars attached to the lipid A region [43]. It provides structural stability to the LPS molecule and connects the lipid A to the O antigen. The composition and structure of the core oligosaccharide can vary between bacterial strains and contribute to the antigenic diversity of LPS.
(iii) O-Antigen: The O antigen is a repeating polysaccharide chain extending from the core oligosaccharide. It consists of various sugar residues arranged in a specific pattern, which is unique to each bacterial strain. The O antigen is highly antigenic and plays a crucial role in the serological classification of Gram-negative bacteria. It is also involved in host–pathogen interactions and can affect the virulence of bacterial strains [44].

The sugars and lipid structures of LPS are essential for the structural integrity, immunogenicity, and biological activity of these molecules. Variations in these structures contribute to the diversity of LPS among different bacterial species and strains, influencing their pathogenicity and host immune responses.

## 6. Origins of LPS in Blood

Sites of normal flora are commonly the source of LPS, including the skin, mucous membranes, and gut microbiota, in addition to any bacterial infection [45]. Disintegration of the bacterial cell releases endotoxins that may cross the gastrointestinal barrier and enter the bloodstream, leading to endotoxemia. A healthy human body has mechanisms to prevent endotoxemia [46] from occurring. However, in individuals with damaged gut tissue, high lipid concentrations, or gastrointestinal diseases, these protective barriers can be compromised, allowing LPS to enter the bloodstream and potentially cause severe effects. The process by which LPS moves from the gut lumen into the systemic circulation remains uncertain. LPS may cross the intestinal epithelium via a transcellular pathway [47]. It was reported earlier that a high-fat diet increases blood LPS levels due to enhanced LPS influx from the gut, which is suppressed by oral administration of intestinal alkaline phosphatase, an LPS-inactivating enzyme [48]. An increase in LPS is linked to changes in intestinal microbiota, with diet-induced dysbiosis potentially contributing to metabolic endotoxemia. Studies in animals and humans show varying impacts on gut microbiota based on diet fat [49,50]. In conditions like Alcoholic liver disease, the intestinal barrier can weaken, leading to much higher concentrations of blood LPS compared to healthy individuals [51].

Absorbed LPS produced by gut bacteria may enter the bloodstream. This is especially harmful if it reaches the basal side of the intestinal tissue, as this is the site where more critical internal tissues and organs can be exposed to the toxin in the body. If high concentrations of LPS travel through the portal vein (the primary source of blood for the liver) and activate TLR4 receptors on hepatic cells including resident Kupffer cells, it can cause systemic inflammation. Studies have shown that during exposure to long-chain fatty acids, LPS is transported from the mucosal to the serosal side of the small intestine by CD36 and other lipid-mediated mechanisms [52]. During absorption, LPS is quickly taken into the portal vein via transcytosis pathways within villous cells, but it enters the lymphatic system at a slower rate. This mechanism is dependent upon the chylomicron-mediated absorption pathway directly to the lymph. Under physiological conditions, transcellular pathways are used more than paracellular pathways [52]. Chronic exposure to low levels of LPS can lead to complications beyond septic shock and endotoxemia. This condition, known as metabolic endotoxemia, occurs when LPS from gut bacteria leaks into the bloodstream due to a compromised gut barrier. Low-grade inflammation associated with metabolic endotoxemia has been implicated in various diseases, including diabetes, obesity, non-alcoholic fatty liver diseases, chronic kidney disease, fibromyalgia, chronic fatigue, and cardiovascular disease [53,54]. It has also been linked to immune disruption in HIV [55]. Metabolic endotoxemia is commonly observed in inflammatory bowel diseases [56].

## 7. LPS Clearance by Liver

While the inflammatory response to LPS is widespread, the liver is the primary organ involved in LPS systemic clearance. It has been demonstrated that the liver clears about 80% of rough LPS administered intravenously [4,6,57]. Coulthard et al. have demonstrated two phases in the clearance of both smooth and rough LPS [58]. A rapid clearance that happens in 5 min and a slow clearance that takes place in hours. The clearance of LPS in the liver is thought to be mediated exclusively by Kupffer cells [4,59,60,61] and hepatocytes [9,62,63]. It is understood that macrophages dephosphorylate and deacylate lipid A following phagocytosis [10,11], thereby inactivating LPS. While these enzymatic processes are well understood, macrophage-mediated uptake and modification is gradual. Thus, Kupffer cells and hepatocytes are the primary cells involved in slow LPS clearance. Rapid clearance of LPS from the blood in the order of minutes is performed by LSECs [6]. Using high-resolution microscopy images, it was observed that 75% of infused LPS became associated with LSEC within 2-4 min. Detection of LPS with LSECs lasted around 45 min post-infusion, suggesting that LSECs are major contributors to the rapid early clearance [6].

This is not surprising given the voracious scavenging ability [10] of LSECs for multiple circulating smaller molecules less than 200 nm, including small immune complexes and viruses [64,65] and importantly, the size of LPS is also in the range of 10–50 nm [66]. Furthermore, LSEC are highly specialized for endocytosis, as they feature a range of crucial endocytic receptors. These receptors include mannose receptors, collagen receptors, hyaluronan receptors, L-SIGN, FcγRIIb, and, notably, various scavenger receptors [67,68].

In summary, LSECs are efficient scavengers during the rapid phase of LPS clearance. Although Kupffer cells and hepatocytes offer additional support during the slower phase, the LPS clearance process overall is not entirely effective. The liver’s inability to eliminate excess LPS can lead to systemic inflammation [56,69]. When the sinusoidal cells are injured along with the impaired ability of cells to handle normal LPS from the gut, a marked increase in circulating LPS levels in the blood is observed [70].

## 8. Blood Components That Facilitate Clearance

Previous studies have demonstrated that incubating LPS with serum can alter its clearance or signaling abilities [71]. Significantly, it has been observed that the majority of LPS captured by LBP is quickly sequestered by lipoproteins [72,73]. Over 90% of LPS in blood circulation has been shown to bind to lipoproteins; binding affinities in decreasing order are High Density Lipoprotein (HDL) > Low Density Lipoprotein (LDL) > Very Low Density Lipoprotein (VLDL) [74]. The affinity of LPS for different lipoproteins varies based on their phospholipid content [75,76]. HDL, with its high phospholipid content, has the greatest LPS binding capacity in human plasma [76]. Low plasma HDL-cholesterol levels have been consistently associated with endotoxin-mediated diseases [77,78,79,80] and severe sepsis in clinical studies [78]. Moreover, individuals with low HDL levels exhibit a stronger inflammatory response to LPS [78]. Interestingly, increasing the HDL concentration in mice has been shown to protect against endotoxin challenge, and transgenic mice with elevated HDL are resistant to endotoxin [74,81]. Although the literature has attributed HDL-mediated protection to the neutralization of LPS, the significantly slower rate of LPS from the LPS-LBP-HDL complex compared to the clearance rate of lipoprotein suggests that neutralization may not be the in vivo mechanism by which HDL functions [77,78,79,80]. Yao et al. provide evidence that HDL is the plasma component responsible for transporting LPS to LSECs, thereby aiding in its clearance [6]. In a subsequent study, the authors found that LPS-HDL complexes taken up by LSECs localize to lysosomes [3], indicating a degradative pathway for both LPS and HDL. This finding also helps explain the association between plasma HDL-cholesterol levels and endotoxin-mediated diseases.

Additional studies are needed to clarify whether HDL or other lipoproteins aid in the slow clearance of LPS by Kupffer cells to understand the mechanisms involved, especially since macrophages are the major cell types involved in LPS signaling. Research should focus on how lipoproteins interact with LPS and Kupffer cells, the signaling pathways affecting LPS uptake, and the impact on immune responses and liver function. Comparing the roles of different lipoproteins, such as LDL and VLDL, and exploring the clinical implications of these interactions will also be crucial.

## 9. Receptors Involved in LPS Clearance

Although the liver expresses multiple endocytic scavenger receptors, and LSECs and Kupffer cells are known for their high endocytic activity, the concept of LPS clearance being a receptor-mediated phenomenon was not considered until 1991. The first study to demonstrate in vivo, that scavenger receptors are major players in endotoxin clearance is from Hampton et al. [10]. However, most in vitro research has indicated that scavenger receptors on macrophages play a crucial role in the clearance and detoxification of endotoxin in animals [10]. Recent work by Cabral et al. has identified Stabilin-1 and -2 receptors expressed by LSECs as key players in clearing LPS from the bloodstream. Despite this, the observation that Stabilin receptor double knockout mice can still clear LPS suggests the existence of other scavenger receptors that may also contribute to this process [3].

Other receptors expressed in LSECs and Kupffer cells may also be responsible for the binding and uptake of LPS to varying degrees. Many of these receptors are pattern recognition receptors that are common scavengers for a host of ligands that share common charge/shape characteristics. Past research efforts to identify specific receptors involved competition studies using common ligands such as acetylated low-density lipoprotein (AcLDL) or oxidized LDL (oxLDL) [61,82]. Extensive work performed by van Oosten and co-workers found that scavenger receptor type A (SR-A) which is expressed by both LSECs and Kupffer cells and macrosialin expressed by Kupffer cells had a limited effect on the binding of LPS [83]. Further work by Yamamoto and colleagues demonstrates that LPS inhalation promotes the increase in cells expressing SR-A in the bronchoalveolar lavage fluid suggesting that this receptor is involved with the resolution of LPS exposure within the lung. However, the study never definitively measures LPS binding with SR-A or that SR-A expressing cells were enriched with internalized LPS [84]. More recent studies have confirmed that SR-A binds and internalizes LPS with the participation of CD14, which also prevents the engagement of TLR4, a proinflammatory receptor that is also a known receptor of LPS that stimulates the immune response through signaling, but not for LPS internalization [85]. In a J774 model, blocking antibodies for CD14 inhibited uptake of LPS by up to 70% suggesting that both SR-A and CD14 work in tandem for LPS internalization [38,86,87,88,89,90,91,92,93,94].

Although most of the work in the past 25 years has focused on SR-A and CD14 as scavenger and signaling receptors for LPS, other receptors may also contribute to LPS clearance. SCARA4 of the class A family of scavenger receptors which is also named SRCL, CLP, or COLEC12 has been shown to have strong binding with *E. coli* and *S. Aureus* [95]. Purified SCARA4 bound strongly with LPS using in vitro assays, however, internalization in a cellular or animal model has not been demonstrated [96]. Scavenger receptor B (SR-B1 or CLA-1) is a well-characterized pattern recognition receptor that may also bind LPS [97]. However, that may occur through HDL, which is a molecule that is recognized by both LPS and SR-B1 [98]. CD36 is a common fatty acid binding protein implicated in aiding free fatty acids to cross the plasma membrane. CD36 is also classified as a scavenger receptor in the class B family that is implicated in the mobilization of many different types of molecules [99]. Due to its capability for binding hydrophobic molecules, it may also participate in LPS internalization in a concerted mechanism with other molecules [94,100]. In addition, in longer experiments (24 h), both scavenger receptor-dependent and -independent uptake were demonstrated to be responsible for the lysosomal catabolism of endotoxin [10]. Interestingly, TLR4 in hepatocytes has been shown to be involved in LPS clearance [9] several hours after exposure, suggesting different receptors are involved in early and late phases.

## 10. Intracellular Adaptor Proteins and Vesicles Involved in LPS Clearance

It is highly plausible that a full set of extra and intra-cellular LPS binding proteins participate in collaborating with LPS clearance receptors. Understanding the cellular and molecular mechanisms is vital for comprehending LPS clearance in immunology. This comprehensive knowledge is integral to our understanding of fundamental cellular processes such as receptor-mediated endocytosis, vesicle trafficking, receptor recycling, and intracellular signaling. Importantly, this innovative contribution extends beyond the realm of bacterial infections and endotoxemia, providing valuable insights into diverse cellular processes associated with the management of molecular complexes comprising lipids, sugars, and proteins that are exemplified by lipoproteins and LPS. Previous literature has demonstrated that bactericidal/permeability-increasing protein (BPI) binds to LPS with very high affinity and inhibits LPS activity in the chromogenic Limulus Amoebocyte Lysate Assay (LAL) assay [101,102]. Importantly, BPI was shown to inhibit LPS-induced cell activation in mononuclear phagocytes [103]. The other lipid-binding proteins known to offer survival advantage after LPS administration in mice are Phospholipid transfer protein (PLTP) and cholesteryl ester transfer protein (CETP) [104,105], however, recent data show that CETP has no direct interaction with LPS and does not offer a survival advantage when LPS is given intravenously [106]. The relationship between these serum proteins and LPS clearance receptors is yet to be understood (Figure 2).

The receptor-mediated endocytosis process is facilitated by adapter proteins [107] and during endocytosis, the cargo and its receptors are incorporated into pits/vesicles formed by Clathrin or Caveolin [108]. The adapter protein and vesicle that facilitates the endocytic clearance of LPS is not known. Caveolin-1 (Cav-1) is a crucial protein in caveolae, responsible for their structure and signaling. It is a small protein that promotes membrane curvature and interacts with several other proteins to regulate endocytosis, receptor internalization, cholesterol accumulation, and cell signaling. Recent studies have implicated Cav-1 as a modulator of innate immunity and inflammation [109,110,111]. Mice lacking Cav-1 were more susceptible to Salmonella infection, however, macrophages derived from these mice showed increased inflammation in responses to bacterial LPS [109]. The deletion of Cav-1 suppresses CD14/CD36 expression and TLR4-MyD88-NF-κB signaling in macrophages, leading to impaired phagocytosis and inflammatory cytokine production [112]. Also, Cav-1 has been found to interact with TLR4 and attenuate LPS-induced proinflammatory cytokine production in murine macrophages when stimulated with carbon monoxide [113]. Furthermore, Clathrin-mediated endocytosis is known to be prominent in LSECs, especially during very early time points [114], and could be associated with Stabilin-1 (Stab1) and Stabilin-2 (Stab2) during LPS clearance [3]. Caveolin is known to be involved in lipid sorting [115] and may be involved in sorting LPS as well as for clearance. It is possible that adaptor proteins like GULP (PTB domain-containing engulfment adapter protein) [116] are involved in the clearance of LPS. Previous research indicates that Disabled-2 (Dab2) have negative regulatory effect on TLR4 signaling [117], and may be a potential adaptor molecule involved in LPS clearance via Stabilin receptors and thus functions contrary to TLR4 [3].

## 11. Enzyme Involved in LPS Inactivation

Out of the three structural components of LPS, lipid A is the most bioactive component and generates the endotoxin response. In the lipid-A portion of LPS, the phosphates at the 1′ and 4′ positions of the glucosamine disaccharide backbone and acyl chains play a significant role in its biological activity [44]. LPS degradation by endocytosis pathway terminates in lysosomes (100–500 nm diameter), a small organelle with an acidic environment and containing about 60 different hydrolytic enzymes including proteases and lipases. Previous literature demonstrated that LPS-HDL complexes which undergo a degradative process in LSECs are located within lysosomes after endocytosis [3]. LSECs, likely due to their role in degrading endocytosed waste materials, are expected to have high lysosomal enzymatic activity. Earlier studies conducted in rats demonstrated that LSECs and Kupffer cells, in comparison to parenchymal cells (PCs), exhibit higher specific lysosomal enzyme activities [118,119,120]. In a study by Kjetil H et al., LSECs were found to have higher specific lysosomal enzyme activities than metabolically active PCs, and specific activities of glucuronidase and α-mannosidase were also higher in LSECs than in Kupffer cells [121]. The high lysosomal enzymes in LSECs align with their role as professional scavenger cells. Moreover, studies in rats suggest that LSECs can recruit lysosomal enzymes from the circulation via the mannose receptor [122,123], potentially explaining their high activity in pigs.

In the lipid-A portion of LPS, the phosphates in the 1′ and 4′ positions of the glucosamine disaccharide backbone and acyl chains are implied to have a major role in the bioactivity of LPS [44] (Figure 3). LPS uptake by LSECs can lead to its enzymatic inactivation through two well-characterized mechanisms. One mode of inactivation involves the dephosphorylation of the phosphate groups on the LPS molecule, which reduces its activity and alters its biological effects [10]. Another mechanism is the deacylation of the primary acyl chains of lipid A, a process mediated by the enzyme acyloxyacyl hydrolase (AOAH) [11,124,125]. This enzymatic modification diminishes the endotoxic properties of LPS by modifying its lipid A structure, thereby impacting its interaction with TLR4. Additionally, both mechanisms contribute to the detoxification and clearance of LPS from the circulation, playing crucial roles in maintaining immune homeostasis.

## 12. TLR4 Signaling

Previous studies have established that TLR4 serves as the signaling receptor for LPS. Mice with dysfunctional TLR4 exhibit reduced responsiveness to LPS [126,127] and the expression of TLR4 levels determines the susceptibility to LPS [128]. To prevent systemic activation of immune response by LPS, the liver in a healthy state is known to express TLR4 at minimal levels [129,130].

The role of CD14 in both the internalization and signaling of LPS exposure was first reported in 1992 [87] along with other studies performed in the Munford laboratory [87,88,89] and further characterized by Triantafilou and colleagues [90,91,92,131]. Many of these studies point to a balance between TLR4 and CD14 surface expression that controls uptake and signaling for LPS. Most recently, there is some evidence that CD14 plays a regulatory role for TLR4 by controlling TLR4 exposure or response with LPS [93,94].

Several LPS-binding proteins found in serum and cell membranes [132,133,134] help bridge the connection between LPS and TLR4. Among those, CD14, and LPS-binding protein (LBP) are essential for LPS recognition by TLR4 and MD-2 [132,135,136]. The catalytic process involved in LBP transferring LPS to CD14 has recently been discovered [137]. LBP interacts with LPS micelles and forms short-lived complexes with CD14, both in its secreted (sCD14) and membrane-bound forms (mCD14) [137]. Once the CD14/LBP/LPS complex is formed, CD14 is released from LBP and binds to individual LPS molecules [137]. Transferring LPS to mCD14 increases the sensitivity of TLR4/MD-2 to LPS in innate immune cells like macrophages and monocytes. This is the body’s first defense against bacterial invasion [137,138,139]. In contrast, transferring LPS to sCD14 can trigger a response in cells lacking CD14 [140,141]. In addition to its traditional role of increasing LPS sensitivity, CD14 expressed on the cell surface is essential for the internalization of TLR4 and TRIF (toll/interleukin 1 receptor-domain-containing adapter-inducing interferon-β), leading to signaling from the endosome [142,143]. In CD14-positive cells, like macrophages, TLR4 is internalized into endosomes, where it releases from MyD88 and interacts with TRIF, to activate IRF3 [144,145]. Activated IRF3 is involved in type I interferon production.

It was originally believed that TLR4 located on the cell surface is solely responsible for recognizing LPS and initiating both MyD88-dependent and TRAM–TRIF-dependent pathways [146]. The internalization of LPS/TLR4 complex was thought to be involved in both LPS clearance and TLR4 recycling [147]. However, recent studies have shown that TLR4 recognizes LPS on the cell surface and activates the MyD88-dependent pathway. The internalization of the LPS/TLR4 complex into endosomes triggered the TRAM–TRIF-dependent pathway in macrophages [148]. The internalization of the LPS/TLR4 complex is a receptor-mediated process that may or may not involve clathrin [149]. However, recent studies using siRNA to reduce clathrin expression have shown that the early phase (up to 40 min) of LPS internalization is primarily clathrin-dependent [150].

LPS binding to the TLR4 and MD2 triggers morphological/physical changes in the TLR4’s TIR domain. Unlike other TLRs, TLR4 utilizes both MYD88 [151] and TRIF [152] signaling adaptors, as well as the respective adaptor molecules MyD88-adaptor-like [MAL] [153], which is also known as TIRAP [154] and TRIF-related adaptor molecule [TRAM [155]]. This dual signaling capability enables TLR4 to elicit two distinct responses: rapid pro-inflammatory cytokine production at the plasma membrane and a slower type I interferon response mediated by internalization and TRIF signaling from the endosome [156]. LPS/TLR4 signaling activates NF-kB, resulting in the production of pro-inflammatory cytokines (IL-6, IL-1β, IL-18, and TNF-α), chemokines, and other molecules. The IL-1β and IL-18 are the members of the IL-1 family and are produced in pro form. IL-1β and IL-18 must be cleaved by the enzyme caspase-1 to become mature and catalytically active cytokines. These catalytic events happen in a high-molecular-weight multi-protein complex known as the inflammasome [157]. The most extensively studied inflammasome is the NOD-like receptor pyrin-domain containing 3 (NLRP3) inflammasome which consists of a catalytic sensor, NLRP3, a caspase-recruitment domain (ASC) adaptor molecule, and activated caspase-1. The cellular response to LPS in NLRP3 inflammasome activation has been implicated [158]. However, recently LPS has been shown to prompt the activation of a distinct type of inflammasome, called the non-canonical inflammasome [159,160,161]). The caspase-1-dependent activation of inflammasomes is called canonical inflammasome activation [162], however, the caspase-11 (in mice) or caspase-4/5 (in humans) mediated activation pathway is called the non-canonical inflammasome activation [159,160,161]. Unlike canonical inflammasomes, where multiple protein components are involved in the ligand sensing, assembly, and effector functions, the non-canonical caspases function as both sensor and effector molecules for LPS [163].

## 13. Liver Cells and Their Role in Clearance and Sensitivity to LPS

The major cell types in the liver are hepatocytes and non-parenchymal cells (NPCs). The hepatocytes account for 60–80% of the total cell population [164]. The non-parenchymal cells consist of lymphocytes, dendritic cells (DCs), hepatic stellate cells (HSCs), Kupffer cells, and LSECs.

### 13.1. Hepatocytes

Evidence suggested that primary cultured hepatocytes only respond to TLR2 and TLR4 ligands but express TLR1-9 [165]. Hepatocytes play an important role in the uptake and removal of LPS from the circulation in collaboration with TLR4, CD14, and MD-2 [8,63,166].

### 13.2. Hepatic Stellate Cells (HSCs)

HSCs make up a very small population (<1%) of the total NPCs in the liver. HSCs get activated in the case of liver injury, and activated HSCs produce extracellular matrix (ECM) components such as type 1, 3, and 4 collagen, which leads to the deposition of these ECM components in the liver and ultimately liver fibrosis [167]. Activated human HSCs express CD14 and TLR4, respond to LPS, and secrete pro-inflammatory cytokines such as IL-8 [168].

### 13.3. Kupffer Cells

Kupffer cells are hepatic-resident macrophages and account for about 20% of the NPCs in the liver. Kupffer cells are involved in phagocytosis and antigen presentation, and they are the primary cells that encounter gut-derived toxins such as LPS and mount immune response in the liver [169]. Kupffer cells in the liver and monocytes are crucial immune cells that express TLR4, thus making them sensitive to LPS. These cells also express a high amount of CD14, which is necessary for the inflammatory pathway that LPS brings about [170]. Extremely low concentrations of LPS can activate these immune cells, especially monocytes, and result in them secreting pro-inflammatory cytokines such as tumor necrosis factor a (TNF-α), interleukin IL-6, and IL-1β [171]).

### 13.4. Liver Sinusoidal Endothelial Cells

LSECs comprise the wall of liver sinusoids and account for 15–20% of the total liver cell number and 50% of the NPCs, which is 3% of the total liver volume [172]. LSECs differ from other endothelial cell types and are the best example representing the heterogeneity in structure and function between different endothelial cells [173]. Among all endothelial cells, LSECs are highly specialized cells with discontinuous architecture and are very porous with specialized holes or “fenestrae” to facilitate exposure of blood solutes with the hepatocytes [172]. LSEC fenestration facilitates the transfer of lipoproteins, chylomicron remnants, and macromolecules to the space of Disse, where they are subsequently taken up by hepatocytes [174,175]. The structure and function of LSECs are interconnected, as the defenestration is an early sign of LSEC dysfunction [176]. Unlike other endothelial cells, LSECs are highly efficient at endocytosis and can clear soluble waste macromolecules and colloid materials, including blood-born adenovirus [65,177]. LSECs possess significant activity of scavenger receptors responsible for the clearing of several molecules like advanced glycation end-product (AGE) proteins [178], oxLDL [179]), acLDL [180], hyaluronan [181], chondroitin sulfate [182], and amino-terminal procollagen propeptides [183]. There are 12 different classes in the scavenger receptor family, which are structurally unrelated proteins and have a common affinity for polyanionic molecules [184]. The classification is defined in [185], namely SR-A to SR-L. The LSECs express several of these receptors which belong to the class SR-A (also known as macrophage SR), SR-B (SR-B1 and CD36), SR-E, SR-H (Stabilin-1/FEEL-1/CLEVER-1 and Stabilin-2/FEEL-2/HARE), SR-J, SR-K, and SR-L [186]. LSECs constantly express CD14 and TLR4, which are important for the LPS-inflammatory pathway, but LSECs become LPS tolerant [187] after repetitive stimulation and activation of the receptor’s scavenger activity, and they become refractory to the endotoxin [188].

## 14. LPS Clearance in Human

Cabral et al. showed LPS clearance by murine LSECs that is independent of TLR4 [3]. Similarly, the study by Arias-Alpizar et al. on zebrafish highlights an evolutionarily preserved mechanism for LPS clearance via Stabilin receptors, suggesting that this Stab1 receptor-driven pathway is likely present across vertebrates, including mice and human [189]. Further studies in human liver LSECs and Kupffer cells demonstrating the expression of various scavenger receptors, adapter proteins, and enzymes, in relation to mouse models, are required to understand the pathophysiological effect of endotoxin-associated diseases in humans.

### TLR-4 -LPS Signaling and LPS Clearance Are Interconnected Where One Influences the Other

Although LPS clearance has not been directly studied for its role in LPS signaling, the following studies suggest its involvement. LPS is known to be internalized independent of CD14/TLR4 complex [10,82] and activate caspase-4/5/11mediated non-canonical inflammasome [161]. However, the receptor involved in the internalization of LPS independent of CD14/TLR4 complex is not known. It is plausible that scavenger receptor-mediated uptake of free LPS but not LPS-HDL complexes by LSECs can activate non-canonical inflammasome in liver cells and toll/interleukin 1 receptor-domain-containing adapter-inducing interferon-β/tumor necrosis factor receptor-associated factor (TRIF/TRAF3) mediated IRF3 activation [143,190]. On the contrary, sufficient LPS stimulation has been demonstrated to possess complete inhibition of LPS catabolism. This phenomenon was exhibited at physiologically relevant concentrations of LPS (IC50 less than 1 ng/mL). It was further determined that the inhibition of LPS catabolism is caused by the physiological stimulation of the cells, rather than by competition for uptake, enzymatic sites, or the trapping of labeled lipid IVA within LPS aggregates [8]. On the contrary, Cabral et al. have demonstrated, using KO mouse models of both TLR4 and Stabilin 1 and 2 double knock-outs, that Stabilin receptor endocytoses the LPS or LPS-HDL complex for clearance and degradation, thus it minimizes the access of LPS to TLR4 and limits the TLR4-mediated inflammatory response [3] and thereby, systemic inflammation

## 15. Conclusions

The relationship between LPS clearance and LPS signaling is a dynamic interplay where both processes regulate and influence each other to maintain immune balance. Effective clearance of LPS via Stabilin and other scavenger receptors limits the availability of LPS to activate the TLR4-mediated inflammatory signaling pathways and canonical and non-canonical inflammasome signaling, thereby preventing excessive inflammation. Previous literature has demonstrated that enhancing the efficiency of the LPS clearance process is inversely related to LPS-mediated inflammation; higher clearance by scavenger receptors leads to reduced LPS signaling and inflammation [3].

Conversely, LPS-mediated signaling pathways are speculated to modulate LPS clearance. It is hypothesized that higher levels of LPS signaling impair LPS clearance efficiency, potentially leading to increased inflammatory damage or inhibition of the expression of LPS clearance receptors such as scavenger receptors. It may also be possible that elevated LPS signaling could enhance LPS clearance mechanisms under certain conditions, thus controlling or reducing inflammation. Further research is needed to clarify these processes.

Regarding therapies that specifically target and inhibit LPS signaling, multiple drugs in clinical trials act as pharmacological inhibitors, blocking various signaling proteins. These include TLR4 antagonists, MD2 blockers, compounds that mimic the Lipid A portion of LPS, and inhibitors targeting the TLR4-MyD88 and TLR4-TRIF pathways, etc. The different TLR4 antagonists and small molecule inhibitors of TLR4 have been comprehensively reviewed in these two reviews [190,191]. In addition to these pharmacological agents, anti-inflammatory herbal medicines, such as polysaccharides from *Astragalus membranaceus*, a traditional Chinese herb, have shown promise in suppressing LPS-induced inflammatory responses in a TRIF-dependent manner [192]. Astragaloside IV, a polysaccharide, may inhibit LPS sterically or block TLR4 as a natural anti-inflammatory molecule. Terrain, a fungal metabolite has been demonstrated to protect against LPS-induced endotoxemia by blocking NFkB p65 subunit phosphorylation, thereby blocking inflammatory cytokine production [193].

Alternatively, to enhance LPS clearance mechanisms, a proposed strategy is to increase the expression of Stabilin receptors. For example, Stabilin-1 in circulating monocytes and possibly other immune cells can be upregulated using IL-4/dexamethasone-like drugs that promote the M2 macrophage phenotype. Furthermore, treatment with the recombinant murine proprotein convertase subtilisin/kexin type 9 (PCSK9) has significantly increased the expression of key scavenger receptors such as SRA, CD36, and LOX1, thereby enhancing the uptake of oxidized LDL in TNF-α-primed macrophages [194]. These studies provided proof of the concept that targeting scavenger receptors to modulate immune responses through their expression could be a potential therapeutic strategy. In the context of atherosclerosis and cardiovascular disease (CVD) treatment, the regulation of scavenger receptor expression is a well-studied phenomenon [195], and various therapeutic strategies are under investigation. These include the repurposing of existing clinically approved drugs, the use of herbal remedies, and the exploration of advanced clinical approaches such as, nanoparticle administration and gene therapy [195]. For instance, Pitavastatin, a type of statin drug, has been shown to cause a significant increase in SR-B1 expression and enhance binding to HDL in both murine and human macrophages [196]. However, drugs specifically known to upregulate Stabilin receptors and their functions, or repurposing CVD drugs for this purpose, have not yet been studied.

Rather than concentrating solely on upregulating scavenger receptors, enhancing the expression of molecules involved in LPS clearance could be a more practical strategy. For example, raising HDL levels with recombinant HDL [197] or increasing Apolipoprotein A-I that has been demonstrated to be vital for LBP and HDL interactions [198], might offer a more straightforward solution. Understanding the fundamental mechanisms of LPS clearance by Stabilin and scavenger receptors and identifying all intracellular and extracellular molecules that facilitate this process is crucial for advancing research.

Overall, while LPS signaling blockers are effective, enhancing LPS clearance for therapeutics is more advantageous due to its alignment with host defense mechanisms, which is likely to result in fewer adverse effects. Thus, investigating LPS clearance through various scavenger receptors not only advances our understanding of immune responses but also fosters innovative approaches for combating and managing inflammation during endotoxemia.

## Abbreviations

LPS, lipopolysaccharide; LSECs, liver sinusoidal endothelial cells; TLR4, Toll-like receptor 4; PAMP, pathogen-associated molecular pattern.
